# Potential advantages of a novel chitosan-N-acetylcysteine surface modified nanostructured lipid carrier on the performance of ophthalmic delivery of curcumin

**DOI:** 10.1038/srep28796

**Published:** 2016-06-28

**Authors:** Dandan Liu, Jinyu Li, Hao Pan, Fengwei He, Zhidong Liu, Qingyin Wu, Chunping Bai, Shihui Yu, Xinggang Yang

**Affiliations:** 1 School of Biomedical & Chemical Engineering, Liaoning Institute of Science and Technology, Benxi 117004, PR China; 2 School of Pharmacy, Shenyang Pharmaceutical University, Shenyang 110016, PR China; 3 School of Pharmacy, Queen’s University Belfast, University Road, Belfast BT7 1NN, Northern Ireland, UK; 4 Engineering Research Center of Modern Chinese Medicine Discovery and Preparation Technique, Ministry of Education, Tianjin 300193, PR China

## Abstract

The transient precorneal retention time and low penetration capacity into intraocular tissues are the key obstacles that hinder the ophthalmic drug delivery of many therapeutic compounds, especially for drugs with poor solubility and permeability. To break the stalemate, N-acetyl-L-cysteine functionalized chitosan copolymer (CS-NAC), which exhibit marked bioadhesion and permeation enhancing effect, was synthesized. The curcumin encapsulated NLC (CUR-NLC) was produced and optimized followed by surface absorption of CS-NAC. After coating, changed particle size from 50.76 ± 2.21 nm to 88.64 ± 1.25 nm and reversed zeta potential from −20.38 ± 0.39 mV to 22.51 ± 0.34 mV was observed. The *in vitro* CUR release from NLC was slower than that of CUR-NLC and chitosan hydrochlorides (CH) coated NLC due to the inter and/or intramolecular disulfide formation of thiomers on the surface of nanocarriers. The modification also significantly enhanced transcorneal penetration compared with CH-NLC and the uncoated ones. The effect on bioadhesion and precorneal retention were evaluated by *in vivo* imaging technique and ocular pharmacokinetics studies revealing that the clearance of the formulations was significantly delayed in the presence of CS-NAC and the effect was positively related to the degree of thiolation. In summary, CS-NAC-NLC presented a series of notable advantages for ophthalmic drug application.

Topical drug delivery into eyes is generally regarded as the most convenient and efficient route for the treatment of various eye diseases. Most pharmaceutical preparations are administrated to eyes to treat eyeball surface or intraocular disorders. Due to the limited area and time for absorption in the eye, the drugs for ophthalmic delivery shows low bioavailability, and less than 5% of drug reaches intraocular tissues^1^. Meanwhile, the systemic absorption from the conjunctival sac and the corneal epithelial barrier also limits drug absorption. In addition, the eye has many protective mechanisms, including blinking reflex, lachrymal secretion and nasolacrimal drainage^2^. Therefore, frequent instillation of eye drops are often required to get the expected therapeutic efficacy, which may lead to discomfort and a decrease in patient compliance, especially in long-term therapy.

In the recent years, many research efforts have been made to develop drug delivery systems that would prolong the pre-ocular retention and promote the absorption of drugs^3^,^4^,^5^. Nanostructured lipid carriers (NLC), a second generation of solid lipid nanoparticles (SLN), based on mixture of solid lipids with spatially incompatible liquid lipids combines many features beneficial for ocular application such as controlled drug release, high drug loading, good bioavailability and excellent tolerability^6^,^7^. Nevertheless, the precorneal retention time of NLC is still needed to be improved due to the rapid removal by tears and the undesirable permeability across the corneal epithelium.

Thiolated polymers (thiomers) are polymers with thiol group-containing side-chains, which has been established as a promising new class of polymeric excipients^8^,^9^. Thiolated chitosan (TCS), the most commonly used thiomers, is a readily soluble polymer that can be synthetized by coupling free thiol agents with the amino groups of chitosan (CS). In contrast with CS, TCS has many predominant features. First, TCS have shown strongly improved residence time based on forming covalent (disulphide) bonds with cysteine-rich subdomains of the mucus layer^10^. Second, thiomers have characteristics of permeation enhancement through the reversible opening of the tight junction, enzyme inhibition and efflux pump inhibition^11^,^12^. The mechanism involves electrostatic interaction between positively charged TCS and negatively charged sites in the tight junctions, which results in drug transport via transiently opened tight junctions. Third, solutions of TCS display *in situ* gelling properties at physiological pH values^13^. Consequently, the specialties of TCS ensure the improvement in drug residence time and bioavailability.

Curcumin (CUR) is a yellow polyphenolic compound derived from the rhizome of the plant Curcuma longa, which has been extensively investigated on its anti-inflammatory, anti-microbial, anti-oxidant, and anti-cancer effects^14^,^15^,^16^. As for ophthalmic application, CUR is a potential candidate to treat cornea and retina neovascularization^17^, chronic anterior uveitis^18^, and inhibit the proliferation of lens epithelial cells^3^. However, the insolubility of CUR and the inherent penetration barriers in cornea make it difficult for CUR to enter eyes.

Consequently, the aim of our study was to investigate whether the thiolated NLC could improve the precorneal retention time and cornea penetration of the CUR-NLC to achieve a better ophthalmic performance. In this study, TCS was firstly synthesized by covalent modification of N-acetyl-L-cysteine (NAC) with CS. Then, CUR loaded NLC was developed, optimized and surface modified with CS-NAC. The physicochemical characteristics of the developed formulations were evaluated. The *in vitro* release, *ex vivo* permeability and *in vivo* ocular irritation test were finally carried out to further investigate the surface modification and the degree of thiolation on the efficiency of CUR ocular delivery.

## Results and Discussion

### Synthesis and characterization of the CS-NAC conjugate

**Figure 1:**
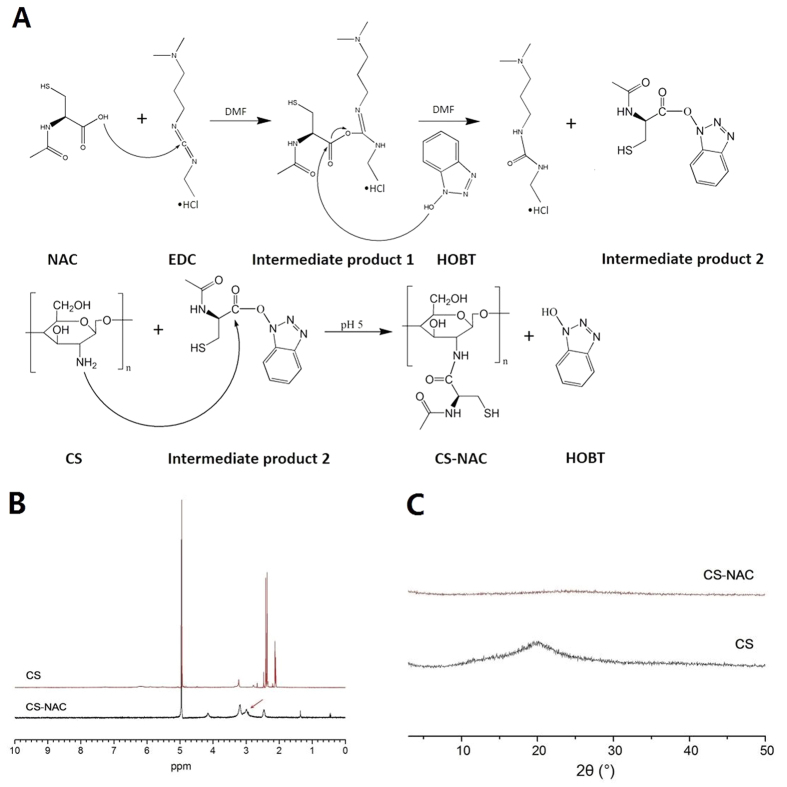
(**A**) Synthetic scheme of CS-NAC copolymer. (**B**) ^1^H NMR spectrum of CS and CS-NAC copolymer. (**C**) PXRD patterns of CS and CS-NAC.

In this study, CS-NAC with low, medium and high degree of substitution (CS-NAC_L_, CS-NAC_M_, and CS-NAC_H_) was synthesized following literature with modification^19^. The covalent linkage of NAC to CS was achieved by formation of amide bonds between the primary amino groups of the polymer and the carboxylic acid group of NAC. The general scheme of the reaction is shown in Fig. 1A. In our study, N, N-dimethylformamide (DMF) was selected as the reaction medium rather than water in contrast with the previously reported study, and the reaction was split into two steps. This was because the intermediate product 1 was readily hydrolyzing in water, which would decrease the productivity of the activated NAC (intermediate product 2). Thus the covalent linkage of NAC to CS would be influenced and the content of free sulfhydryl group would be reduced on CS-NAC. Accordingly, the addition of DMF was to prevent the broken of the ester linkage of intermediate product 1 in water, and subsequently made the thiolation more efficiently. After lyophilization, CS-NAC appeared as white, odorless and fibrous powder, which was soluble in aqueous solution. As shown in Fig. 1B, the structure of CS-NAC was confirmed by ^1^H NMR spectra. The proton peak at 2.92 ppm was ascribed to the side-chain methylene (CH_2_SH) of CS-NAC, which indicated that NAC was successfully conjugated to the CS backbone^20^ (For more details, please see the Supplementary Information file).

To study potential changes in crystalline state of CS-NAC, powder X-ray diffraction (PXRD) measurement was performed. As shown in Fig. 1C, the typical wide crystalline peak of CS (20.08°) was detected. In contrast, no trace of the typical crystalline peaks of CS-NAC was observed, implying the noncrystalline state of the conjugate. This was because the intermolecular hydrogen bonds were significantly reduced due to the decrease of the amount of the free amino groups after chemical modification. The phenomenon further implied the formation of the TCS copolymer.

Ellman’s test was used to determine the amount of thiol groups attached to the polymer. As depicted in Table 1, an increase in the NAC: CS molar ratio led to a higher number of thiol groups, and 1 g of CS- NAC copolymer contained about 496.7 μmol of thiol groups at most. It was reported that the free thiol groups could be oxidized to inter- or intramolecular disulfide bond^21^. According to our results, the oxidation process was retarded in our experiment, and more than 82% free thiol groups were maintained.

### Central Composite Design

#### Model fitting and statistical analysis

A total of 20 experiments were conducted to evaluate the influence of CUR-NLC producing parameters on the four responses. Measured response data for all experimental runs of CCD are listed in Table 2. By applying multiple regression analysis, the experimental data were fitted to a quadratic polynomial model and the equations are shown below in the form of coded factors:


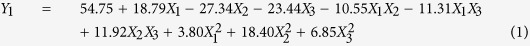



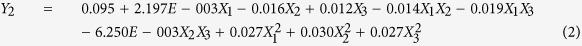



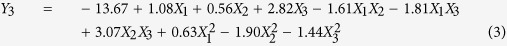



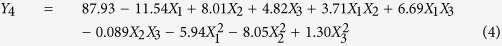


Determination coefficient (R^2^), adjusted determination coefficient (R^2^_adj_), and predicted determination coefficient (R^2^_pred_) were used to estimate the goodness of the fit of the model^22^,^23^. The R^2^ value was 0.9851, 0.9620, 0.9760 and 0.9736, which implied that 98.51% 96.20%, 97.60%, and 97.36% of the variations could be explained by the predicted model. The R^2^_adj_ values of 0.9717, 0.9277, 0.9544, and 0.9499 indicated the high degree of correlation between the observed and predicted values. The R^2^_pred_ values of 0.8968, 0.7305, 0.8315 and 0.8097 were in reasonable agreement with R^2^_adj_. The analysis results demonstrated that the relationship between factors and the responses of the predicted model were well-correlated.

Analysis of variance (ANOVA) of the experimental data was summarized in Table S2 (please see the Supplementary Information file). It confirms the model (quadratic model, *p* < 0.05) obtained for all responses of CUR-NLC, and also provides significant factors affecting these responses. The variables would be more significant if the F-value became greater and the *p*-value became smaller. The model F-values of 73.70, 28.10, 45.21, and 40.99 for the four responses implied that the model was statistically significant, and there was only a 0.01% chance that the “model F-value” was due to the noise. As is shown in Table S2, the total mass of medium chain triglyceride (MCT) and glyceryl monostearate (GMS) (X_1_), the GMS/MCT mass ratio (X_2_), and the quantity of Solutol HS15 (X_3_) were considered significant for the mean particle size (PS, Y_1_), zeta potential (ZP, Y_3_) and entrapment efficiency (EE, Y_4_). For polydispersity index (PI, Y_2_), the GMS/MCT mass ratio and the content of Solutol HS15 were identified significant.

#### Analysis of response surface

**Figure 2:**
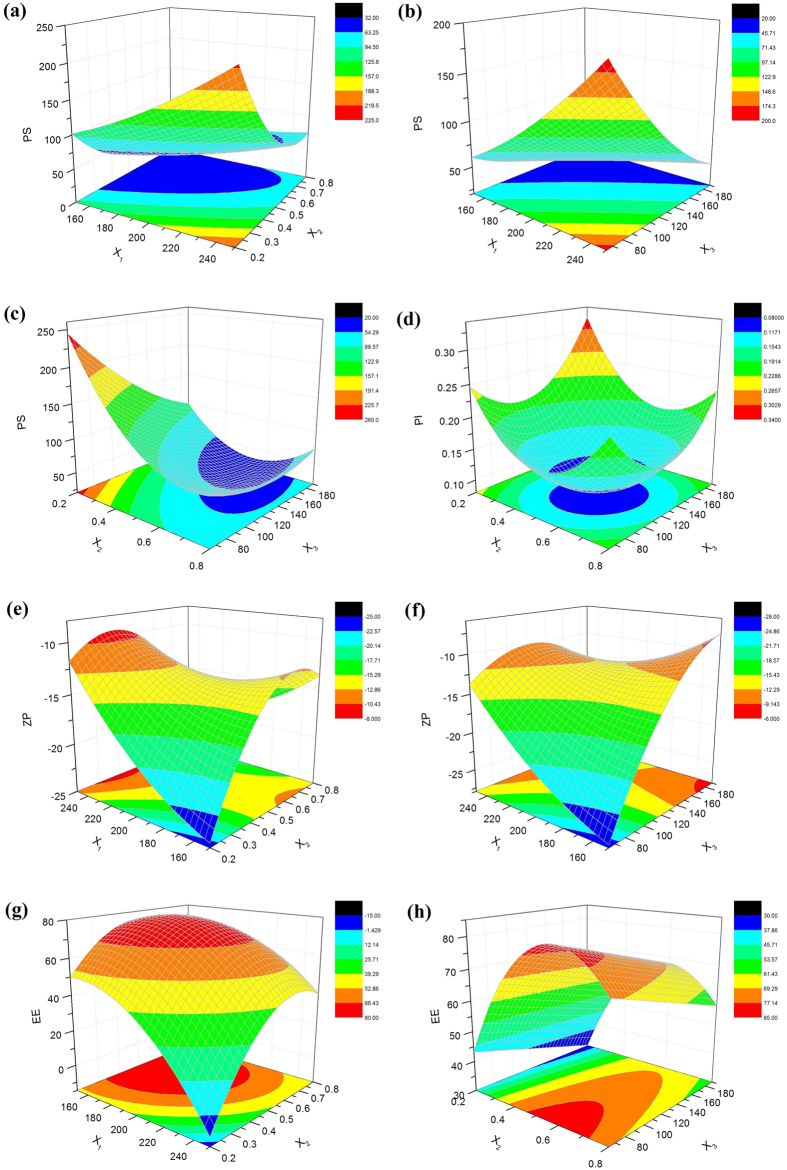
Three-dimensional (3D) response surface plots showing the effect of the variables on the responses.

The three-dimensional response surface plots for the most statistical significant variables on the evaluated parameters are shown in Fig. 2. The two horizontal axes represented any two in the three independent variables, and the remaining independent variable was kept at zero level, simultaneously.

For an optimized formulation designed to ophthalmic delivery, both the PS and the PI should be the lowest as possible in order to improve the patient comfort during administration^24^. As observed in (a) and (b) of Fig. 2, the PS increases with raising the lipid phase concentration (X_1_). This could be attributed to the increased viscosity of the lipid phase, which reduces the diffusion rates of the solute molecules. The increasing of the lipid phase concentration could also enhance the opportunity of the nanoparticles aggregation^25^. In Fig. 2(a,c,d), all the PS and PI show an initial decrease and then increase with increasing X_2_. At low ratio of solid lipid to liquid lipid, PS and PI decreased with increasing the solid lipid concentration. This may occurred due to the excessive amount of liquid lipid would destroy the disorder in the NLC matrices, and thus the stability of the system would be destroyed. Meanwhile, too much liquid lipid enlarge the gap generated by the structural difference between solid and liquid lipids and make the hole loose, thereby the resultant particle slightly large^7^. However, excessive GMS would increase the dispersion viscosity, leading to higher surface tension and thus larger PS and lower size homogeneity^26^. As shown in Fig. 2(b–d), by increasing X_3_, PS and PI decreased due to the drop of the surface tension until it reached a minimum level. Above this optimum point, diffusion layer got thickened because of the excess coverage of the particles by surfactant at the interface, which would decrease the ZP. Hence, the PS and PI would increase due to agglomeration tendency^27^.

The ZP denotes the electrical charge at the NLC surface, being an important parameter that allows predicting the physical stability and mucoadhesive properties about NLC. A pronounced ZP (|ZP| > 20 mV), either positive or negative, could provide sufficient electrostatic repulsion between particles and avoid aggregation^28^. According to Fig. 2(e,f), the ZP gets less negatively by the increase in X_1_, X_2_ or X_3._ As the increment of the three variables all resulted in the decreasing of the MCT content in the NLCs. Thus the phenomenon could be explained that reducing the concentration of the negative charged MCT (containing of free fatty acids) would put the negative charge amount down on the particles in the process of NLC formation^29^.

With respect to EE, a negative effect of X_1_ and X_3_ was observed. And there was an optimal range existed for X_2_. As mentioned above, the increment in lipid amount would increase the viscosity of the NLC and destroy the stability of the system, resulting in the low EE. Introducing of liquid lipids into solid lipids leads to special inner structures of NLC such as imperfect crystallization, amorphous, and multiple O/F/W types resulting in improved and stable drug loading^30^. From Fig. 2(g,h), incorporation of about 40% liquid lipid might be the most appropriate. Beyond a certain concentration range of MCT, the EE would be decreased due to drug leakage. As observed, the amount of HS15 shows negative effects on the EE. The effect could be attributed to the particle surface formed would be too small to adsorb all surfactant molecules when the surfactant level increased, leading to the formation of micelles in solutions reallocating drug from the NLC into the aqueous phase, reducing the value of EE^31^.

#### Formulation optimization

The optimum ranges for each factor were found by selected constraints as follows: X_1_-in range, X_2_-in range, X_3_-minimize, to reduce irritancy; Y_1_-minimize, Y_2_-minimize, Y_3_-minimize, Y_4_-maximize. The optimized formulation that obtained from Design expert 8.0 software for CUR-NLC should be composed of the total mass of MCT and GMS 181 mg, GMS/MCT mass ratio 0.61, and Solutol HS15 89 mg. Table S3 (see the Supplementary Information file) showed that the experimental values of the three batches prepared within the optimum range were very close to the predicted values, with low percentage bias, suggesting that the optimized formulation was reliable and reasonable.

### Characterization of the CUR-NLCs

#### Particle size, zeta potential and EE

CS is a water insoluble linear polysaccharide. Owing to the amino group in its structure, CS showed as a pH responsive polymer which would become soluble after salt formation with hydrochloric acid on the amino-groups. Chitosan hydrochlorides (CH) is usually selected as the ideal candidates for surface modification of colloidal drug delivery systems due to excellent bioadhesiveness and biocompatibility^32^,^33^. Consequently, the superiorities of CS-NAC-NLC was validated by comparing with CH modified ones. The physicochemical properties of the uncoated and coated NLCs were evaluated, and the results are shown in Table 3. The PS of unmodified CUR-NLC was 50.76 ± 2.21 nm, with a negative charge (−20.38 ± 0.39 mV). As expected, the PS values were significantly increased after surface modification (p < 0.05), and the cationic CH or CS-NAC copolymers reduced the electron cloud density surrounding the NLC particle surface, rendering the integral particles positive. The results supported the adsorption of CH or CS-NAC onto the surface of the nanoparticles. As observed in Table 3, ZP of CS-NAC-NLC particles were all lower than that of CH-NLC, which could be attributed to the increment in the thiol groups anchored to the amino groups of CS. Meanwhile, with an increment in the thiol groups on the CS-NAC conjugates, the PS and ZP values of the coated NLCs were all evidently increased. Since the CS polymers with different thiolation degrees possessed similar zeta potentials, such results also indicated higher amount of CS-NAC adsorption. With respect to the size homogeneity, all the NLCs were evenly distributed with the PI values less than 0.2. Furthermore, the EE of different formulations were above 90%, which was attributed to the hydrophobicity of CUR. A significant improvement in the EE was also observed after coating, which might be attributed to the ionic effect between the polymers and the anionic segment in the core particles. Comparing with CH-NLC, further improvement on EE was observed for CS-NAC modified ones, which may due to the formation of disulfide bonds in or outside the polymers^34^, so that the NLCs become much tighter, thus efficiently prevent the drug escape from the nanoparticles.

#### Morphological studies

**Figure 3:**
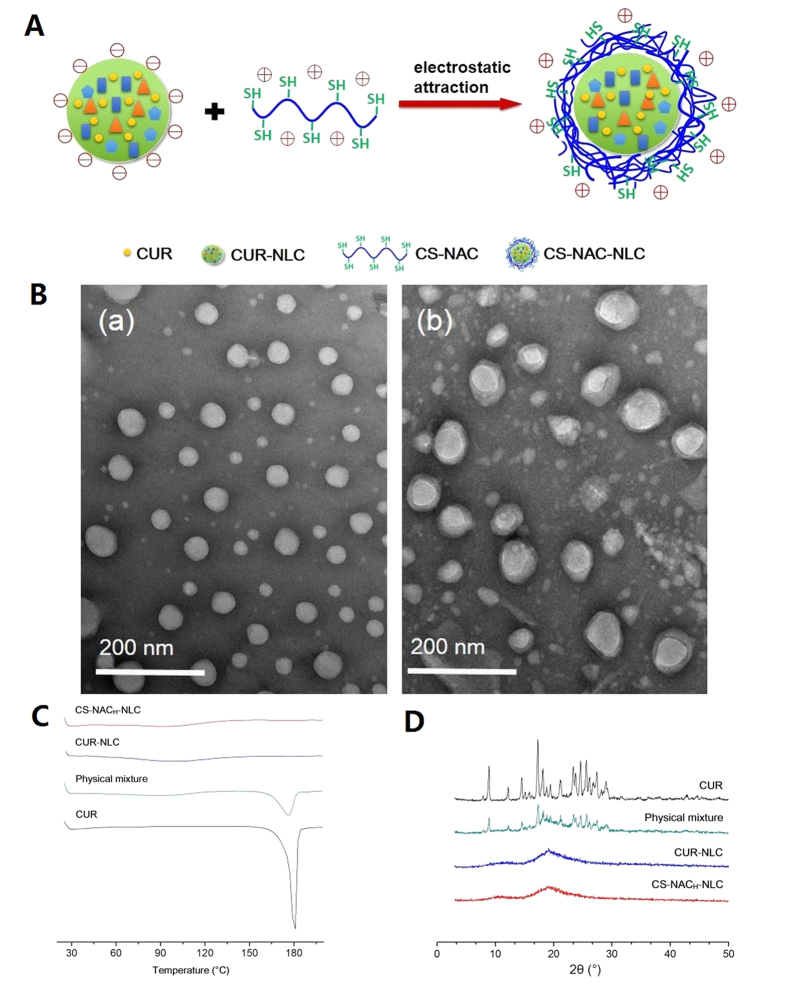
(**A**) Schematic representation of formation of CS-NAC-NLC. (**B**) TEM images of (a) CUR-NLC and (b) CS-NAC_H_ coated CUR-NLC. (**C**) DSC profiles of bulk CUR, physical mixture, CUR-NLC, and CS-NAC_H_-NLC. (**D**) PXRD diffractions of bulk CUR, physical mixture, CUR-NLC, and CS-NAC_H_-NLC.

The NLCs were surface decorated with CS-NAC on the hypothesis that the cationic polymers would be absorbed onto the negative NLC surfaces by electrostatic attraction (Fig. 3A). The TEM images showed that CUR-NLC exhibited a spherical structure and about 50 nm in diameter (Fig. 3B(a)), while the CS-NAC_H_ coated NLC showed larger spherical-shaped particles covered by an outer-layer structure (Fig. 3B(b)). The results efficiently proved our hypothesis, and was also consistent with the above PS analysis.

#### Crystal form of CUR-NLCs

Differential scanning calorimetry (DSC) and PXRD measurements were performed to characterize the physical status of CUR present in NLCs. As shown in the DSC patterns (Fig. 3C), a single sharp endothermic melting peak of CUR at 178.2 °C owing to its melting indicated its crystalline nature. For the physical mixture, the melting peak for CUR also appeared. However, no melting peaks of CUR around 178.2 °C were detected in the DSC curves obtained from the lyophilized CUR-NLC and CS-NAC_H_-NLC, indicating that CUR was encapsulated in the NLCs in a noncrystalline state. To further confirm the physical state of CUR, PXRD analysis was performed. As shown in Fig. 3D, typical diffraction peaks of CUR were visible between 5° and 30°, and these were also observed in the patterns obtained for the physical mixture. However, no trace of the typical crystalline peaks of CUR was observed for the NLCs, further proving the noncrystalline state of drug presented in the NLCs.

#### *In vitro* release

**Figure 4:**
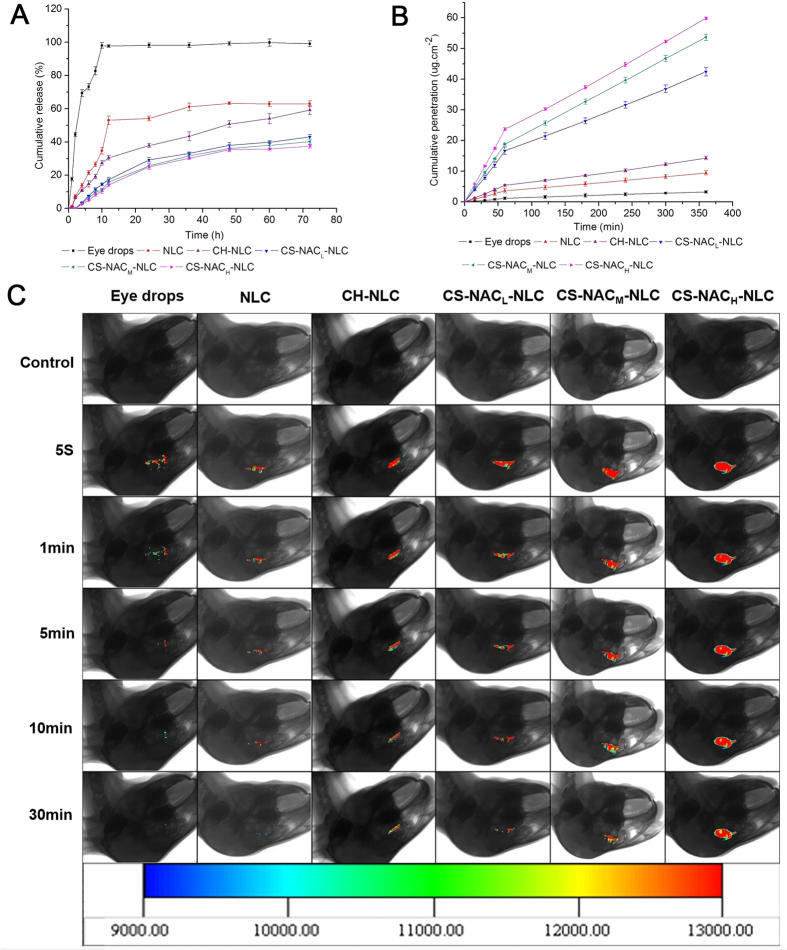
(**A**) *In vitro* cumulative release profiles of CUR preparations. (**B**) *Ex vivo* cornea penetration curves of CUR preparations. (**C**) Real-time *in vivo* fluorescence imaging of CUR preparations.

The dissolution profiles of CUR eye drops and various NLCs are shown in Fig. 4A. In the case of CUR eye drops, over 40% of CUR was dissolved within 2 h, and nearly 100% of drug was released after 12 h. By contrast, all CUR-NLCs presented a biphasic and controlled release manner within 72 h. The phenomenon could be explained by the difference in structure and melting points between solid lipids and liquid lipids. In general, the NLC particles were formed firstly by the solid lipid (with free or little liquid lipid) which owns higher melting point, and followed by the location of most of the liquid lipid and surfactant at the outer shell. The biphasic drug release is therefore due to the diffusion of CUR from the core slower than that from the outer shell. In addition, the drug release rate further decreased after surface modification, especially when modified with CS-NAC. And the content of thiol groups on the CS-NAC copolymer was found to have no obvious effect on the drug release. Before released into the medium, CUR had to go through the nanostructure core and 3D network structure imparted by the cross-linking of CS-NAC, which obviously slowed down the release rate from the coated NLCs. Overall, CS-NAC coated NLCs performed a sustained release character which was beneficial to persistent ocular therapy.

#### *In vitro* corneal permeation

The *ex vivo* cornea penetration study was carried out in order to evaluate the effect of CS-NAC on the drug transcorneal permeability. Figure 4B displays the corneal penetration profiles of CUR eye drops and NLCs. A straight broken line with a turning point at 60 min was obtained for all preparations in the time range from 0 to 360 min, while the corresponding apparent permeability coefficients (P_app_) and R^2^ (correlation coefficient) are displayed in Table 4. As observed, the penetration curves were linear before and after the turning point in all cases (R^2^ > 0.9953), which confirmed that the cornea integrity was maintained throughout the experiments. In addition, all the preparations delivered CUR following a perfect zero-order kinetics and the reduction of penetration rate was observed at 60 min. One possible reason for the phenomenon was that, within 60 min, CUR was transported from the cornea epithelium into the hydrophilic stroma gradually, and a drug reservoir was formed in the stroma, which acts as diffusion barrier to highly lipophilic drugs owing to the hydrophilic nature of the stroma^35^. After 60 min, the concentration of CUR in stroma already got enough so as to impede drugs to pass through stroma. Thus, the P_app_ of all preparations were decreased rapidly in the latter half of the experiments, and this behavior led to a lower J_ss_. Compared with CUR eye drops, CUR-NLC showed a dramatically higher P_app_ due to the presence of the lipid matrix, which easily adheres to and is biocompatible with the corneal epithelial cells. When the NLC was surface modified with CH or CS-NAC, the P_app_ further increased compared with the uncoated ones. This would be explained that the positively charged coated NLC particles persuaded transient loosening of tight junctions among corneal epithelial cells through electrostatic interaction with negatively charged constituents in them^36^. And the adherence effect was also strengthened by the electrostatic force^37^. As for CS-NAC coated ones, the most significant increment on P_app_ and J_ss_ was achieved. This could be attributed to the following aspects. On one hand, thiolation could additionally promote opening of tight junctions through inhibition of protein tyrosine phosphatase^8^. Therefore, drug transport via the paracellular route could be enhanced. On the other hand, CS-NAC displayed excellent mucoadhesive properties because they were shown to interact with cysteine-rich residues of mucus glycoproteins thereby forming disulfide bridges. Thus, the coated NLCs can provide an intimate contact of the polymer with the corneal mucosa and a prolonged residence time on it. Meanwhile, a higher drug concentration gradient offered at the absorption sites facilitated the drug transport. Taking the amount of thiol groups into account, the maximum P_app_ and J_ss_ were obtained for the CS-NAC_H_-NLC group, further proving that thiolation facilitates the transcornea passage of CUR-NLC by opening the tight junctions or by strengthen mucoadhesive force through formation of stable covalent disulfide bonds between the thiol groups of CS-NAC and the cysteine groups of cornea mucin.

#### *Ex vivo* fluorescence imaging study

*In vivo* imaging technology was selected to further evaluate the CS-NAC coating of the NLCs on bioadhesion and precorneal retention ability of the ophthalmic formulations. As shown in Fig. 4C, the fluorescence intensity of the CUR eye drops was the lowest and nearly vanished within 5 min. Although the CUR-NLC could more easily penetrate through the cornea than the eye drops, the rapid precorneal elimination was also observed. In contrast, CH or CS-NAC coated NLCs exhibited good spreading and enhanced retention on the cornea during the investigated time period, which was beneficial for the transcornea passage. But the fluorescence intensity of the CH-NLC and CS-NAC_L_-NLC still got weakened over time rapidly. The most significant improvement in ocular retention was observed for the CS-NAC_H_-NLC group. The results demonstrated that the absorption of CS-NAC onto the surface of NLC was an effective way to prolong the retention time and improve the bioavailability of the nanoparticles. And the effect was positively related to the amount of thiol groups on the thiolated CS.

#### Ocular pharmacokinetics

**Figure 5:**
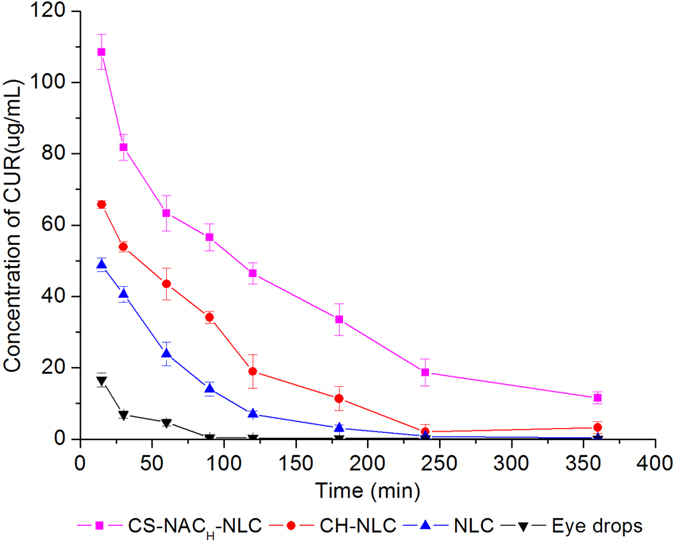
The concentration–time curves of CUR in rabbit tears following topical administration of CUR eye drops and NLCs (mean ± S.D., n = 6).

Pharmacokinetic studies was designed to evaluate the potential of CS-NAC-NLC in the terms of prolong residence at the target site (cornea), and predict the potential of drug from the formulations permeated through the corneal *in vivo*. An ideal formulation should exhibit higher peak tear concentration (C_max_), area under curve (AUC) and mean residence time (MRT) for efficient and prolonged ocular drug exposure. According to Fig. 5 and Table 5, the *in vivo* pharmacokinetic parameters of the four formulations were all found to follow up the following sequence: eye drops < NLC < CH-NLC < CS-NAC_H_-NLC. In contrast with CUR eye drops, NLC, CH-NLC and CS-NAC_H_-NLC got significantly higher C_max_ (3.88-, 5.28-, and 8.88-fold), AUC_0-∞_ (5.97-, 12.25-, and 29.88-fold) and MRT_0-∞_ (1.27-, 2.19-, and 2.91-fold) (all *p* < 0.05). The results were in accordance with that observed *ex vivo* fluorescence imaging studies. Although surface modification of NLC was an effective way to further prolong the ocular retention time and improve the bioavailability of the nanoparticles, the comparative results also revealed that the effectiveness of CH and CS-NAC_H_ were in different level. Even though CH-NLC particles carried more positive charges, thus the ionically interaction between CH-NLC and the negatively charged mucus layer of eye surface should be stronger than that with CS-NAC_H_-NLC, CS-NAC_H_-NLC still has very obvious advantages. This was probably due to the formation of disulfide bonds between the thiol groups on the surface of CS-NAC_H_-NLC particles and the sticky protein in the mucus. In contrast, the ionic interaction, as a non-covalent bond, could only provide weak mucoadhesion, in many cases insufficient to guarantee the localization of a drug delivery system at a given target site. In conclusion, the results proved that CS-NAC coating facilitated the formulation sustainably retained in the precornea, offering a long-last action to increase the drug permeability and thus improve the bioavailability.

## Materials and Methods

### Materials

CUR, CS (Mw = 179.17 kDa, deacetylation degree ≥95%, viscosity = 100–200 mPa·s), NAC and Ellman’s reagent (DTNB, 5,50-dithiobis (2-nitrobenzoic acid)) were all obtained from Aladdin (Shanghai, China). 1-Ethyl-3-(3-dimethylaminopropyl) carbodiimide hydrochloride (EDC·HCl) and 1-Hydroxybenzotrizole (HOBT) were purchased from Medpep Co., Ltd. (Shanghai, China). DMF was obtained from Shanghai Chemical Co., Ltd. (Shanghai, China). GMS was obtained from TianJin Bodi Chemical Holding Co., Ltd. (Tianjin, China). Miglyol 812 N (medium chain triglyceride, MCT)) was obtained from Sasol (Witten, Germany). Solutol HS15 (polyoxyethylene esters of 12-hydroxystearic acid) was supplied by BASF (Lud-wigshafen, Germany). Gelucire 44/14 was kindly gifted by Gattefosse (Paris, France). Rhodamine B was purchased from Sinopharm chemical reagent Co., Ltd. (Shanghai, China). Purified water was used after deionization and filtration. All other chemicals and reagents used were of analytical grade or better.

### Animals

New Zealand albino rabbits (half male and half female, weighting 2.0–2.5 kg) free of any ocular damage were provided by the Lab Animal Center of Shenyang Pharmaceutical University (Shenyang, China). All animal studies were conducted in accordance with the Principles of Laboratory Animal Care, and approved by Shenyang Pharmaceutical University Animal Ethical Committee. The ethical committee approval number of animal studies is SYPU-IACUC-2015-1111-401.

### Synthesis of CS derivatives

In brief, NAC, EDC·HCl and HOBT at a molar ratio of 4:1:1 were dissolved in 8 ml of DMF under continuous stirring for 3 h to activate carboxyl groups of NAC. Meanwhile, 1 g of CS was dissolved in 20% (v/v) HCl solution under stirring to obtain a 1.25% (w/v) polymer solution. Thereafter, dropped the activated NAC into the CS solution at molar ratio of 4:1, 2:1 or 1:1 (NAC: CS). The pH was then adjusted to 5.0 by adding NaOH (1 M), and the reaction was carried out at room temperature under stirring for 3 h without exposure to light. To eliminate the unreacted NAC residues, the reaction mixture was dialyzed at 4 °C in darkness, first against 5 mM HCl, twice against 5 mM HCl containing 1% NaCl, and then two times against 1 mM HCl (12 hrs each time). Finally, the aqueous polymer solution was prefrozen in the refrigerator at −80 °C for 12 hours and subsequently lyophilized using a freeze-drier (Bio Cool, Beijing, China) at −30 °C for 18 hours. Samples were stored at 4 °C for further use. As for the control polymer, namely chitosan hydrochlorides (CH), was prepared in the same way without submitting NAC to the coupling reaction.

### Nuclear magnetic resonance spectroscopy (NMR)

The chemical structure of CS-NAC was identify by proton nuclear magnetic resonance (^1^H NMR) spectra. The ^1^H NMR spectrum were recorded on a Bruker spectrometer operated at a frequency of 400 MHz at room temperature. The CD_2_COOF_3_/D_2_O (1:10, v/v) mixture and D_2_O were selected as the solvents for CS and CS-NAC conjugates, respectively.

### Determination of the thiol group content

The amount of free thiol groups immobilized on CS-NAC conjugate was quantified using Ellman’s test as described previously^38^,^39^. Briefly, 5 mg of CS-NAC polymer was hydrated in 2 mL of deionized water. Then, the test solution was prepared by mixing 100 μL of the polymer solution, 900 μL of 0.5 M phosphate buffer (PBS, pH 8.0) and 1 ml of Ellman’s reagent (3 mg in 10 mL of 0.5 M PBS, pH 8.0). After incubation at room temperature without light for 2 h, the reactant was centrifuged at 4000 rpm for 10 min. The absorbance of the supernatant was measured at a wavelength of 450 nm with a UV-T6 spectrophotometer (Persee Analytic Instrument Co., Ltd., Beijing, China). The amount of thiol groups was calculated from a calibration curve of NAC in a concentration range of 0.125–1.25 mM made in exactly the same way as the samples.

The total amount of thiol groups fixed on the conjugate is a composition of free thiol groups and oxidized thiol groups in form of disulfide bonds. After reduction of disulfide bonds with sodium borohydride (NaBH_4_), the reaction with Ellman’s reagent was carried out to determine the total amount of thiol groups. And the quantity of disulfide bonds could be calculated by subtracting the amount of free thiol groups from the total amount of thiol groups on the polymer^10^,^40^. In brief, after hydrating 1 mg of the polymer with 700 μl deionized water, 300 μl of 0.05 M PBS (pH 6.8) and 2 mL of freshly made 4% NaBH_4_ solution (w/v) were added. The samples were incubated in a water bath at 37 °C for 1 h. Then, 300 μL of 5 M HCl was added to the reaction solution to destroy the remaining NaBH_4_. Then, the solution was neutralized by adding 1 mL of 1 M PBS (pH 8.5). Thereafter, 1 mL of Ellman’s reagent (40 mg in 10 ml of 0.5 M PBS, pH 8.0) was added and the mixtures were agitated for 2 h at room temperature at a dark place. The absorbance was determined at a wavelength of 450 nm with a UV-T6 spectrophotometer. The total amount of thiol groups was calculated from a standard curve of NAC.

### Preparation of the formulations

#### Preparation of CUR loaded NLC

In the present study, CUR-loaded NLC was prepared by melt emulsification technique. GMS and MCT were respectively selected as the solid lipid and liquid lipid whereas Solutol HS15 and Gelucire 44/14 were used as surface active agents. Among them, GMS, MCT and Gelucire 44/14 are all generally recognized as safe (GRAS) compounds^41^,^42^,^43^. Solutol HS15 was chosen due to its physiological compatibility for ophthalmic applications^44^,^45^. Briefly, CUR (6 mg), GMS, MCT and Solutol HS15 were mixed and melted under moderate stirring at 75 °C to form a transparent and uniform oil phase. Then, Gelucire 44/14 (10 mg) was dissolved in 10 mL of deionized water before heated up to 75 °C and added dropwise to the oil phase, with magnetic stirring at 600 rpm for 5 min. The resultant solution was rapidly solidified in an ice bath (0 °C) to form CUR-NLC.

#### Preparation of surface-modified NLC

To obtain NLC surface-modified with CH or CS-NAC, the polymer solution (1 mg/mL) was added dropwise to the same volume of CUR-NLC suspension, followed by a 30 min incubation under continuous agitation at room temperature.

#### Preparation of CUR eye drops

CUR eye drops was prepared by dissolving 3 mg CUR in 10 mL of 15% propylene glycol.

All the preparation process was carried out in an aseptic room under aseptic condition, and the preparations were all sterilized by filtration through a 0.22 μm filter^46^.

#### Central Composite Design

Based on the results of preliminary experiments, a three factor, five level center central composite design (CCD) was utilized to evaluate the formulation factors that affect the PS (Y_1_), PI (Y_2_), ZP (Y_3_), and EE (Y_4_) of CUR-NLC, *i.e.*, the total mass of MCT and GMS (X_1_), GMS/MCT mass ratio (X_2_) and the amount of Solutol HS 15 (X_3_), respectively. The experiments were designed by Design-Expert 8.0 software. Table S4 shows the corresponding CCD in the present study and the experiments were completely randomized.

The responses obtained for this study were well modeled by quadratic functions, as approximated by the equations as follows:


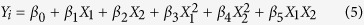


where Y_i_ represents the predicted response, X represents the independent variable, and β represents the coefficient. F-test was used to evaluate lack-of-fit. The nominal of which *p* > 0.05 were selectively deleted for model simplifying within each equation.

#### Mean particle size and zeta potential

The PS, PI, and ZP of the colloidal systems were determined by photon correlation spectroscopy using a Zeta-sizer Nano-ZS-90 (Malvern Instruments Ltd., Worcestershire, UK) at 25 °C. All measurements were performed in triplicate.

#### Drug encapsulation efficiency

EE of CUR-NLC or CS-NAC-NLC was determined by ultrafiltration centrifugation method. First, 0.5 mL of the NLC sample was placed in the upper chamber of a centrifuge tube matched with an ultrafilter (Amicon ultra, MWCO 10 kDa, Millipore Co., Billerica MA, USA) and centrifuged for 15 min at 4000 rpm. In the ultrafiltrate, the unentrapped drug was obtained. The solid residue was redispersed in 10 ml of acetonitrile and filtered through a 0.22 μm membrane filter for assessment in triplicate. To determine the total drug content in NLC, 0.5 mL of the NLC sample was diluted appropriately with acetonitrile. After centrifugation (4000 rpm, 30 min), the supernatant was collected for the determination of CUR concentration. The amount of CUR in the samples was determined by high-performance liquid chromatography (HPLC) using a Diamonsil C18 column (250 mm × 4.6 mm, 5 μm, Dikma, China). The mobile phase was a mixture of acetonitrile and 0.5% phosphoric acid aqueous solution (56:44, v/v). The flow rate was 1.0 mL/min and the column temperature was 25 °C. The wavelength was set at 425 nm. The EE could be calculated using the following equations:


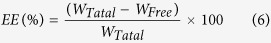


where W_Total_ and W_Free_ are the total weight of CUR in NLC and the unentrapped drug in the ultrafiltrate, respectively.

#### Transmission electron microscopy (TEM) analysis

The morphology of the nanoparticles was observed by TEM (JEM-1200EX JEOL, Tokyo, Japan), using a negative-staining method. Samples were prepared by drying a dispersion of the nanoparticles, diluted 30-fold with deionized water, on a copper grid coated with an amorphous carbon film. After being negatively stained with 2% phosphotungstic acid and air-dried under room temperature, the samples were completed for observation.

#### PXRD analysis

The status of CUR in NLCs was analyzed using a DX-2700 micro-diffractometer (Aolong Radiative Instrument Group Co., Ltd., Dandong, China). The date were recorded under graphite monochromatized Cu Kα radiation over the 2θ range from 3° to 50° at 40 kV and 40 mA.

#### DSC analysis

DSC analysis was performed using a DSC 1 calorimeter (Mettler Toledo, Schwerzenbach, Switzerland). 5 mg of the samples were scanned at a heating rate of 10 °C/min under nitrogen over a temperature range of 25 °C–200 °C in an aluminum pan and sealed hermetically, using an empty pan as the reference.

#### *In vitro* release study

Dissolution tests of the preparations were evaluated using dynamic dialysis method. Before the test, accurately weighed samples containing 0.3 mg CUR was placed into a dialysis bag (molecular weight cut off 8000–14,000) and fixed on the stirring paddle of the USP Apparatus 2 setup (ZRS-6G, TiandaTianfa Technology Co., Ltd, Tianjin, China). The tests were performed at 34 ± 0.5 °C in 250 mL of the release medium (PBS with 1.2% Tween-80, pH 7.4) with paddle speed set at 100 rpm. The samples (2 mL) were withdrawn, and the same volume of fresh dissolution medium were added to the system at predetermined time intervals and filtered through a 0.22 μm filter membrane. The drug content was analyzed by the HPLC method described above. Each releasing experiment was performed in triplicate.

#### Permeability of drug through the isolated-cornea

The corneal penetration evaluation was carried out in isolated rabbit corneas (available areas 0.5 cm^2^) using Franz-type cells (Tian Mei Da Instruments, China). First, the rabbit corneas were excised, weighed and then stored in an iced (4 °C) Glutathione bicarbonate Ringer’s (GBR) buffer immediately. The preparations (equivalent to 0.3 mg CUR) and 4 mL GBR solution containing 1.2% Tween80 were applied into the epithelial (donor) side and endothelial (receptor) side of the cornea, respectively. The apparatus were maintained at 34 ± 0.5 °C. At scheduled time intervals, 0.5 mL sample was withdrawn from the receiving compartment, and was immediately replaced with an equal volume of preheated diffusion medium. Each experiment was continued for 6 h in triplicate. The amount of CUR released into the receptor phase was analyzed by the HPLC method described above. The cumulative penetration quantity at various intervals was calculated as follows^37^:


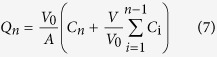


where V_0_ and V indicate the volume of the dissolution medium and the sample, respectively; C_n_ stands for the drug concentration of the dissolution media at each sampling time; C_i_ is the drug concentration of the ith sample, and A is the penetrating region area (0.5 cm^2^).

The rate of drug penetration was measured by the apparent permeability coefficient (P_app_) and J_ss_ (steady-state flux) as follows:


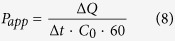



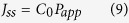


where C_0_ is the original concentration of drug in the donor chamber, 60 is the conversion of units from minute to second, ΔQ/Δt refers to the slope rate of the straight line portion on Q_n_–t plot and A is the penetrating region area (0.5 cm^2^).

#### Precorneal retention evaluation of NLCs using *ex vivo* imaging technology

Precorneal retention time of the preparations was assessed using the *in vivo* imaging technology. To label the NLCs with fluorescein, 3 mg of rhodamine B was added to the aqueous phase during NLCs preparation process, and the oil phase was prepared without the addition of CUR. The following process was the same as aforementioned. For the labeled eye drops, 3 mg of rhodamine B was totally dissolved in 10 mL of deionized water. The five formulations without rhodamine B were selected as control, respectively. Before imaging, one drop of the preparation was instilled onto the right cornea of the anesthetized rabbits. Then the rabbits were detected in head using the *in vivo* imaging system (Carestream image station system FX Pro Care stream Health, Inc., USA) equipped with filter sets (excitation/emission, 530/600 nm)^5^,^47^.

#### Ocular pharmacokinetics

Twenty four rabbits were divided into four groups comprising six animals in each. The rabbits were fed a standard pellet diet with free access to water. In brief, 200 μL of formulation, corresponding to 0.3 mg of CUR, was instilled into the lower conjunctival sac of the eye (n = 6), and the eyes were closed manually for 10 s, then the tear samples were collected at the time intervals of 15, 30, 60, 90, 120, 180, 240, 300 and 360 min after the formulation instilled. The untreated contralateral eyes were used as control and 0.9% (w/v) NaCl solution was instilled into the control eye. The collection of tear was performed by gently inserting a dry weighted filter paper strip (2 mm × 5 mm) into the lower eyelid of the rabbit and keeping the strip staying for 10s under the eyes closed manually. The strip soaked with tear was weighted, and the weight gain pre and post-sampling was recorded to calculate the amount of tear collected. Then the strip was put into a centrifuge tube, and 200 μL of acetonitrile was added into the tube for extracting CUR from the strip by vortex for 90 s, then the tube was centrifuged at 14,000 rpm for 15 min^48^. The concentration of CUR in the supernatant was determined with HPLC.

#### Statistical analysis

Statistical analysis was performed using Student’s t-test and differences were judged to be significant at *p* < 0.05.

## Additional Information

**How to cite this article**: Liu, D. *et al*. Potential advantages of a novel chitosan-N-acetylcysteine surface modified nanostructured lipid carrier on the performance of ophthalmic delivery of curcumin. *Sci. Rep.* **6**, 28796; doi: 10.1038/srep28796 (2016).

## Supplementary Material

Supplementary Information

## Figures and Tables

**Table 1:** The content of thiol groups attached to the CS (mean ± S.D., n = 3).

Copolymer	NAC:CS (molar ratio)	Free thiol groups (μmol/g)	Disulfide content (μmol/g)	Total thiol groups (μmol/g)
CS-NAC_H_	4:1	496.7 ± 17.1	103.5 ± 19.4	600.2 ± 28.8
CS-NAC_M_	2:1	320.3 ± 25.1	69.6 ± 5.3	389.9 ± 22.5
CS-NAC_L_	1:1	200.3 ± 17.3	43.6 ± 16.9	243.9 ± 15.4

**Table 2:** Factor levels and observed responses for central composite design.

No.	Levels of independent factors			Responses			
	X_1_ (mg)	X_2_	X_3_ (mg)	Y_1_(nm)	Y_2_	Y_3_(mV)	Y_4_(%)
1	150	0.50	127	43.37 ± 0.87	0.18 ± 0.02	−14.4 ± 0.32	90.87 ± 1.23
2	170	0.32	90	99.74 ± 2.32	0.13 ± 0.01	−20.4 ± 0.56	84.11 ± 0.78
3	170	0.68	164	41.61 ± 1.78	0.19 ± 0.00	−8.01 ± 0.21	88.79 ± 2.03
4	170	0.68	90	49.59 ± 2.78	0.14 ± 0.01	−22.90 ± 0.45	88.96 ± 1.15
5	170	0.32	164	52.30 ± 3.48	0.22 ± 0.03	−17.64 ± 0.67	83.21 ± 1.59
6	200	0.50	190	39.53 ± 2.54	0.19 ± 0.02	−12.20 ± 0.28	97.19 ± 3.03
7	200	0.80	127	57.77 ± 1.56	0.17 ± 0.01	−16.93 ± 0.33	83.93 ± 1.45
8	200	0.50	127	56.56 ± 3.87	0.09 ± 0.01	−13.62 ± 0.71	87.79 ± 0.54
9	200	0.50	64	110.14 ± 5.02	0.17 ± 0.03	−22.76 ± 0.68	88.57 ± 1.02
10	200	0.50	127	57.87 ± 1.69	0.10 ± 0.00	−13.18 ± 0.15	88.31 ± 0.44
11	200	0.20	127	157.23 ± 6.18	0.21 ± 0.01	−20.63 ± 0.37	48.9 ± 1.67
12	200	0.50	127	52.36 ± 1.34	0.09 ± 0.02	−13.51 ± 0.43	87.74 ± 0.60
13	200	0.50	127	58.47 ± 2.77	0.10 ± 0.01	−12.93 ± 0.28	85.21 ± 0.39
14	200	0.50	127	53.28 ± 0.99	0.09 ± 0.03	−13.47 ± 0.22	85.44 ± 1.59
15	200	0.50	127	49.74 ± 3.11	0.10 ± 0.02	−14.15 ± 0.41	88.93 ± 1.65
16	230	0.68	164	46.96 ± 2.55	0.14 ± 0.03	−13.43 ± 0.18	85.20 ± 0.74
17	230	0.32	90	192.52 ± 4.03	0.21 ± 0.01	−12.12 ± 0.35	38.93 ± 0.70
18	230	0.32	164	91.65 ± 3.87	0.21 ± 0.04	−16.75 ± 0.17	65.87 ± 0.83
19	230	0.68	90	91.97 ± 1.21	0.15 ± 0.02	−21.21 ± 0.09	59.70 ± 1.22
20	250	0.50	127	89.03 ± 0.85	0.18 ± 0.01	−8.88 ± 0.06	53.89 ± 0.99

Factors—X_1_: the total mass of medium chain triglyceride (MCT) and glyceryl monostearate (GMS); X_2_: GMS/MCT mass ratio; and X_3_: the amount of Solutol HS15. Responses—Y_1_: the mean particle size (PS); Y_2_: polydispersity index (PI); Y_3_: zeta potential (ZP); and Y_4_: entrapment efficiency (EE).

**Table 3:** Physicochemical characterization of NLCs (mean ± S.D., n = 3).

Responses	NLC	CH-NLC	CS-NAC_H_-NLC	CS-NAC_M_-NLC	CS-NAC_L_-NLC
PS (nm)	50.76 ± 2.21	93.04 ± 1.87^a^	88.64 ± 1.25^a^,^b^	71.52 ± 1.43^a^,^c^	70.25 ± 1.81^a^,^c^
PI	0.11 ± 0.02	0.28 ± 0.05^a^	0.17 ± 0.01^a^,^b^	0.16 ± 0.02^a^,^b^	0.16 ± 0.02^a^,^b^
ZP (mV)	−20.38 ± 0.39	30.23 ± 0.25^a^	22.51 ± 0.34^a^,^b^	14.62 ± 0.64^a^,^b^,^c^	11.73 ± 0.27^a^,^b^,^c^
EE (%)	90.06 ± 1.82	90.49 ± 3.21^a^	96.62 ± 3.13^a^,^b^	95.33 ± 2.25^a^,^b^	95.02 ± 0.81^a^,^b^

^a^Statistically significant compared with the NLC (p < 0.05).

^b^Statistically significant compared with the CH-NLC (p < 0.05).

^c^Statistically significant compared with the CS-NAC_H_-NLC (p < 0.05).

**Table 4:** Permeation parameters of CUR formulations through the excised corneas (mean ± S.D., n = 3).

	Eye drops	NLC	CH-NLC	CS-NAC_L_-NLC	CS-NAC_M_-NLC	CS-NAC_H_-NLC
J_ss (0–60 min)_ (×10^−4^ μg·cm^−2^·s^−1^)	3.20 ± 0.35	10.13 ± 0.21^a^	15.3 ± 0.33^a^,^b^	46.06 ± 0.15^a^,^b^,^c^	57.65 ± 0.61^a^,^b^,^c^,^d^	65.56 ± 0.64^a^,^b^,^c^,^d^,^e^
J_ss (60–360 min)_ (×10^−4^ μg·cm^−2^·s^−1^)	1.66 ± 0.07	3.30 ± 0.06^a^	5.12 ± 0.03^a^,^b^	14.98 ± 0.07^a^,^b^,^c^	20.05 ± 0.06^a^,^b^,^c^,^d^	21.4 ± 0.34^a^,^b^,^c^,^d^
P_app (0–60 min)_ (×10^−6^ cm·s^−1^)	0.54 ± 0.04	1.69 ± 0.09^a^	2.55 ± 0.02^a^,^b^	7.67 ± 0.11^a^,^b^,^c^	8.71 ± 0.06^a^,^b^,^c^,^d^	10.90 ± 0.21^a^,^b^,^c^,^d^,^e^
P_app (60–360 min)_ (×10^−6^ cm·s^−1^)	0.19 ± 0.01	0.56 ± 0.02^a^	0.85 ± 0.02^a^,^b^	2.99 ± 0.12^a^,^b^,^c^	3.35 ± 0.10^a^,^b^,^c^,^d^	3.57 ± 0.05^a^,^b^,^c^,^d^
R^2^_(0–60 min)_	0.9954 ± 0.0017	0.9994 ± 0.0005	0.9998 ± 0.0004	0.9986 ± 0.0012	1.0000 ± 0.0003	0.9998 ± 0.0002
R^2^_(60–360 min)_	0.9981 ± 0.0023	0.9997 ± 0.0008	0.9953 ± 0.0001	1.0000 ± 0.0002	0.9999 ± 0.0013	0.9999 ± 0.0011

^a^Statistically significant compared with the eye drops (p < 0.05).

^b^Statistically significant compared with the NLC (p < 0.05).

^c^Statistically significant compared with the CH-NLC (p < 0.05).

^d^Statistically significant compared with the CS-NAC_L_-NLC (p < 0.05).

^e^Statistically significant compared with the CS-NAC_M_-NLC (p < 0.05).

**Table 5:** Pharmacokinetic parameters of CUR eye drops and NLCs in rabbit tears (mean ± S.D., n = 6).

Group	C_max_ (μg/mL)	AUC_(0-∞)_ (μg/mL/min)	MRT_(0-∞)_(min)
Eye drops	16.58 ± 1.98	606.95 ± 25.23	48.42 ± 10.20
NLC	64.36 ± 2.25^a^	3622.32 ± 59.51^a^	61.73 ± 1.79^a^
CH-NLC	87.62 ± 2.68^a^,^b^	7435.69 ± 379.39^a^,^b^	106.21 ± 16.51^a^,^b^
CS-NAC_H_-NLC	147.36 ± 4.93^a^,^b^,^c^	18134.61 ± 863.72^a^,^b^,^c^	141.14 ± 15.45^a^,^b^,^c^

^a^Statistically significant compared with the eye drops (p < 0.05).

^b^Statistically significant compared with the NLC (p < 0.05).

^c^Statistically significant compared with the CH-NLC (p < 0.05).
