# Supplementary material for: Potential advantages of a novel chitosan-N-acetylcysteine surface modified nanostructured lipid carrier on the performance of ophthalmic delivery of curcumin
Source: Sci Rep. 2016 Jun 28;6:28796. doi: 10.1038/srep28796 (PMC4923878; doi:10.1038/srep28796)
Supplement: Supplementary Information [file srep28796-s1.pdf]

Potential advantages of a novel chitosan-N-acetylcysteine surface modified nanostructured lipid carrier on the performance of ophthalmic delivery of curcumin

Supplementary Information

Dandan Liu[1,*], Jinyu Li[2], Hao Pan[3,*], Fengwei He[1], Zhidong Liu[4], Qingyin Wu[1], Chunping Bai[1], Shihui Yu[2], Xinggang Yang[2]

[1]School of Biomedical & Chemical Engineering, Liaoning Institute of Science and Technology, Benxi 117004, PR China;

[2]School of Pharmacy, Shenyang Pharmaceutical University, Shenyang 110016, PR China;

[3]School of Pharmacy, Queen's University Belfast, University Road, Belfast BT7 1NN, Northern Ireland, UK;

[4]Engineering Research Center of Modern Chinese Medicine Discovery and Preparation Technique, Ministry of Education, Tianjin 300193, PR China.

*Correspondence and requests for materials should be addressed to D.L. (email: liudandan1124@126.com) or H.P. (email: pwstfzy@126.com)

Results and discussion

Synthesis and characterization of the CS-NAC conjugate.

In our study, the combination of EDC·HCl and HOBt could effectively guarantee the productivity of the activated NAC (intermediate product 2), thus improved the yield of CS-NAC conjugates significantly. Consequently, the influence of NAC, EDC·HCl and HOBT molar ratio on the amount of thiol groups fixed on the copolymer was investigated. As depicted in Table S1 (code 1-4 and 8-10), an increase in the EDC·HCl or HOBT molar ratio has little impact on the number of thiol groups. This might be because excess EDC·HCl or HOBT lead to a heterogeneous reaction system. But the number of immobilized thiol groups was strongly dependent on the molar ratio of NAC (Table S1, code 1 and 5-7). The content of free thiol groups nearly reached maximum when the NAC, EDC·HCl and HOBT ratio was 4:1:1, and there was no further significant increase in coupling beyond this level. Therefore, the molar ratio of NAC, EDC·HCl and HOBT was fixed at 4:1:1.

Code	CS:NAC:HOBT : EDC·HCl (molar ratio)	Free thiol groups (μmol/g)	Disulfide content (μmol/g)	Total thiol groups (μmol/g)
1	1:1:1:1	177.2±22.4	48.0±14.5	225.2±12.4
2	1:1:1:2	169.0±12.5	55.3±12.2	224.3±11.1
3	1:1:1:4	184.4±29.6	46.1±12.6	230.5±17.4
4	1:1:1:8	183.1±28.7	62.7±14.1	245.8±15.3
5	1:2:1:1	290.6±10.4	62.3±15.9	352.8±26.3
6	1:4:1:1	496.7±17.1	103.5±19.4	600.2±28.8
7	1:8:1:1	470.9±7.33	166.3±20.1	637.2±32.6
8	1:4:2:1	497.3±16.2	103.3±22.1	600.6±19.8
9	1:4:4:1	495.4±16.8	112.3±23.7	607.7±18.7
10	1:4:8:1	478.3±11.5	122.6±25.4	600.9±32.8

Table S1. Influence of NAC, HOBT and EDC·HCl molar ratio on the amount of thiol groups fixed on the copolymer.

Source	DF[a]	Y_1		Y_2		Y_3		Y_4	
		F-value[b]	*p*-value[c]	F-value[b]	*p*-value[c]	F-value[b]	*p*-value[c]	F-value[b]	*p*-value[c]
Model	9	73.40	< 0.0001*	28.10	< 0.0001*	45.21	< 0.0001*	40.99	< 0.0001*
X_1	1	103.25	< 0.0001*	0.43	0.5264	19.07	0.0014*	136.96	< 0.0001*
X_2	1	218.49	< 0.0001*	22.60	0.0008*	5.06	0.0483*	66.08	< 0.0001*
X_3	1	160.56	< 0.0001*	12.82	0.0050*	130.81	< 0.0001*	23.93	0.0006*
X_1X_2	1	19.06	0.0014*	9.89	0.0104*	24.99	0.0005*	8.29	0.0164*
X_1X_3	1	21.90	0.0009*	18.39	0.0016*	31.58	0.0002*	26.97	0.0004*
X_2X_3	1	24.31	0.0006*	2.04	0.1834	90.45	< 0.0001*	4.747E-003	0.9464
X_1^2	1	4.44	0.0612	68.16	< 0.0001*	6.78	0.0263*	38.35	0.0001*
X_2^2	1	104.41	< 0.0001*	87.26	< 0.0001*	62.41	< 0.0001*	70.40	< 0.0001*
X_3^2	1	14.47	0.0035*	68.16	< 0.0001*	35.85	0.0001*	1.85	0.2039
Lack-of-it	5	6.81	0.0276*	9.20	0.0147*	9.83	0.0127*	14.86	0.0051*

Table S2. ANOVA of response surface quadratic model.

X_1: the total mass of medium chain triglyceride (MCT) and glyceryl monostearate (GMS); X_2: GMS/MCT mass ratio; and X_3: the amount of Solutol HS15; Y_1: the mean particle size (PS); Y_2: polydispersity index (PI); Y_3: zeta potential (ZP); and Y_4: entrapment efficiency (EE).

[a]DF, degree of freedom.

[b]F-value, test for comparing model variance with residual variance.

[c]*p*-value, probability of seeing the observed F-value if the null hypothesis is true.

*Significant model terms ($p < 0.05$).

Responses	Predicted value	Observed value	Bias (%)
Y_1 (nm)	54.22	50.76	-6.38
Y_2	0.12	0.11	-8.33
Y_3 (mV)	-21.14	-20.38	-3.60
Y_4 (%)	94.18	90.06	-4.37

Table S3. Comparison of the observed and predicted values under predicted optimum conditions.

Responses—Y_1: PS; Y_2: PI; Y_3: ZP; and Y_4: EE;

Bias (%) = (actual value-predicted value)/predicted value×100;

Observed values: avg., n = 3.

Variables	Range and levels				
	**-1.682**	**-1**	**0**	**1**	**1.682**
X_1 (mg)	150	170	200	230	250
X_2	0.2	0.32	0.5	0.68	0.8
X_3 (mg)	64	90	127	164	190

Table S4. Independent variables and their levels investigated in the CCD.

X_1: the total mass of MCT and GMS; X_2: GMS/MCT mass ratio; and X_3: the amount of Solutol HS15

Ocular irritation test. Fig. S1 presents the histopathological images of the rabbit eyeballs tested with various preparations to investigate their influence on cell structure and tissue integrity. As observed, cell structure and epithelium integrity were maintained after administration of saline, CH-NLC and CS-NAC_H-NLC. The normal cornea structure consisted of three parts, epithelium layer, stroma layer and thin endothelium layer. A typical stratified epithelial layer can be recognized by the appearance of a bulge at the nuclei of the basal columnar cells and the squamous surface cells. Cornea was mainly possessed by stroma containing smooth and parallel fibrous tissues. When treated with CUR eye drops (containing 15% propylene glycol), corneal epithelium got thickened slightly with increased number of epithelial cells and enlarged cell body. The fibrous tissues in stroma were arranged in disorder and infiltrated by inflammatory cells. In contrast to the normal saline groups, the conjunctiva and iris exposed to the eye drops, CH-NLC and CS-NAC_H-NLC showed no pathological changes. No abnormality in the size and location of conjunctival lymphoid tissue appeared in all the conjunctivas. Normal levels and integration of polymorphonuclear cells were observed in the iris. The above results demonstrated the excellent corneal biocompatibility of CS-NAC_H-NLC.

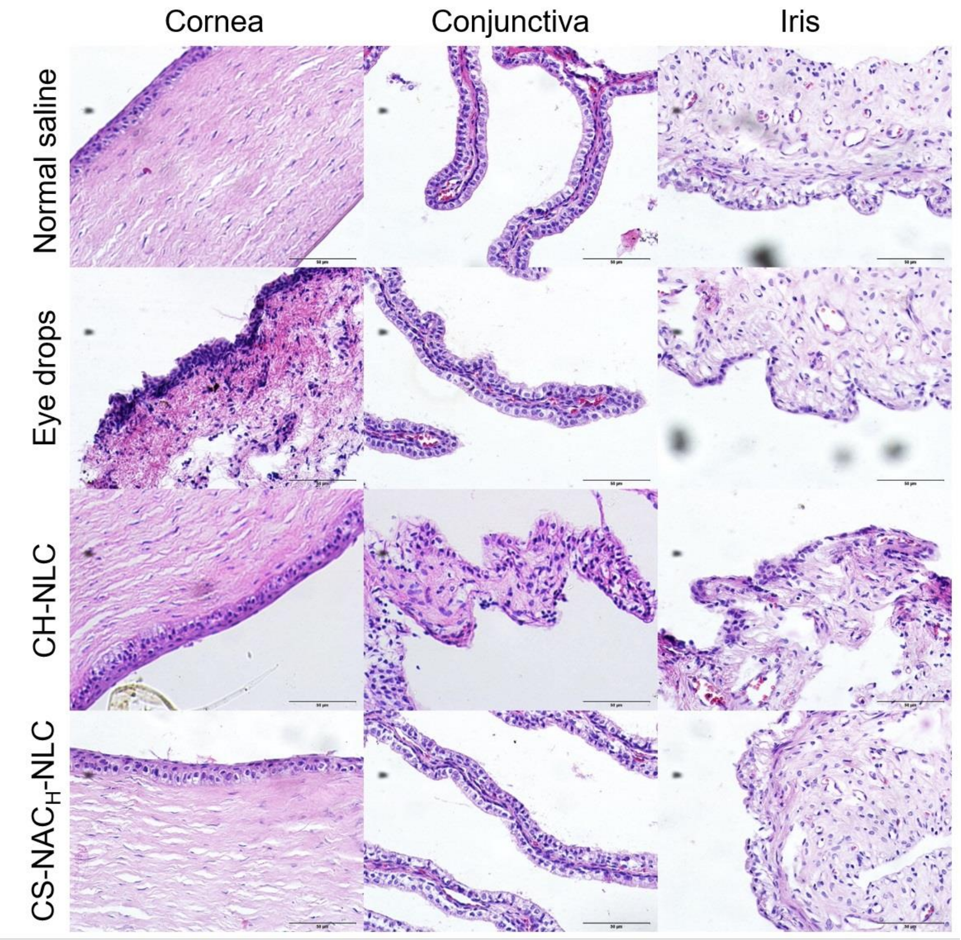

Figure S1. Histopathology microscopy of the ocular tissues including cornea, conjunctiva, and iris after treatment with CUR preparations for 7 days.

Materials and methods

Ocular irritation test. The ocular irritation study was carried out on New Zealand white albino rabbits, which were checked to ensure they had normal eyes before the test. CH or CS-NAC_H modified CUR-NLC and CUR eye drops (50 μL) were instilled into the lower conjunctival sac of the right eyes 5 times a day for 7 days, and the left eyes administrated with the same volume of normal saline served as control. Two hours after the last administration, the rabbits were euthanized by air embolism, and the eye tissues (cornea, conjunctiva, and iris) were fixed in 10% formaldehyde, embedded in paraffin, followed by the preparation of histological sections for viewing.
